# Control of acromegaly in more than 90% of patients after 10 years of pegvisomant therapy: an European referral centre real-life experience

**DOI:** 10.1007/s40618-022-01980-7

**Published:** 2023-03-09

**Authors:** R. Pirchio, R. S. Auriemma, M. E. Montini, A. Vergura, R. Pivonello, A. Colao

**Affiliations:** 1 Dipartimento di Medicina Clinica e Chirurgia, Sezione di Endocrinologia, Università “Federico II” di Napoli, Via S. Pansini 5, 80131 Naples, Italy; 2 Unesco Chair for Health Education and Sustainable Development, “Federico II” University, Naples, Italy

**Keywords:** Acromegaly, Pegvisomant, Long term, Metabolism, Insulin

## Abstract

**Purpose:**

Pegvisomant (PEG) efficaciously controls IGF-I excess in acromegaly and possesses a positive impact on glucose metabolism. Data on very prolonged PEG treatment are still limited, therefore, we investigated the effects of 10-years PEG on disease control, maximal tumour diameter (MTD), and metabolic profile in consecutive patients resistant to somatostatin analogues (SRLs) followed in an European referral centre for acromegaly.

**Methods:**

Since the 2000s, we collected data on anthropometric, hormonal and metabolic parameters, and MTD of patients receiving PEG. In the current study, we included 45 patients (19 men, 26 women, 46.8 ± 11 years) treated for at least 5 years with PEG mono or combined therapy, analyzing data before, after 5- and 10-years PEG.

**Results:**

After10 years, 91% of patients showed full disease control and in 37% a significant decrease in MTD was found. Diabetes prevalence was slightly increased, whereas HbA1c remained stable over the decade. Transaminases remained stable and no case of cutaneous lipohypertrophy was recorded. A different metabolic impact between mono- or combined therapy was found. Patients in monotherapy showed significantly lower fasting glucose (*p* = 0.01), fasting insulin (*p* = 0.008), HbA1c (*p* = 0.007), HOMA-IR (*p* = 0.001), and significantly higher ISI_0_ (*p* = 0.002), whereas patients under combined therapy showed significantly lower total (*p* = 0.03), and LDL cholesterol (*p* = 0.007). Acromegaly duration before PEG was inversely related to ΔFG (*r* = − 0.46, *p* = 0.03) and ΔFI (*r* = − 0.54, *p* = 0.05).

**Conclusions:**

PEG is effective and safe in long term. In patients resistant to SRLs, early beginning of PEG allows a wider gluco-insulinemic improvement.

## Introduction

Acromegaly is a rare disease caused by a pituitary GH-secreting tumour in most cases [1]. Management of acromegaly is multidimensional and treatment is highly personalized [1]. Trans-sphenoidal adenomectomy and somatostatin analogues (i.e. octreotide and lanreotide, SRLs) are cornerstones in treatment of GH- secreting pituitary tumours, although in several patients they are not sufficient to achieve disease control [1]. Indeed, about 25% of patients has been found to be resistant or poorly responsive to SRLs [2].

In these patients, pegvisomant (PEG) represents an excellent therapeutic strategy [1, 3]. PEG is a human GH-receptor antagonist, which competes with endogenous GH at the receptor level; the binding of PEG with the GH-receptor is not followed by its activation, thus determining the interruption of the hepatic production of IGF-I [3]. PEG is approved as a second- or third- line therapy, mainly in patients not cured after neurosurgery or in those who show a poor response to first- line SRLs [1, 3].

PEG monotherapy has been proven to normalize IGF-I levels in about 90% of patients in clinical trials and near to 70% of patients in real- world studies [1, 4–6]. In patients partially responsive to SRLs, the addition of PEG allows to achieve the biochemical control of the disease in most cases [1, 5, 7–9]. Furthermore, recent studies have demonstrated that long-term (i.e. up to 10 years) PEG therapy improves disease control over time, both in monotherapy and in combination with SRLs [10–17]. Concerning long-term PEG efficacy, the analysis of data deriving from ACROSTUDY [11, 17], a global non-interventional surveillance study of long-term treatment with PEG, agree with the results of other previous studies on smaller populations [10, 12–16]. About pituitary adenoma, in most cases the tumour dimensions remain unchanged or even decreased during PEG therapy, mainly when combined with SRLs [8]. According to a recent metanalysis, increase in pituitary tumour volume has been shown to occur in approximately 7% of patients during PEG therapy [6]. This increase in size seems to be mostly ascribed to SRLs withdrawal or to the natural history of an aggressive tumour, rather than a direct effect of PEG therapy [17]. Noteworthy, the incidence of pituitary tumour growth based on local MRI readings has been proven to be higher than that based on central readings [17], suggesting an overestimation of the event in some studies.

Metabolic complications represent a frequent comorbidity in patients with acromegaly [18, 19]. Metabolism disorders associated with acromegaly include insulin resistance, with consequent glucose intolerance till to overt diabetes mellitus (DM), hypertriglyceridemia and decrease in HDL cholesterol [18, 19]. Disease control allows to improve metabolic state and, furthermore, several medical treatment for acromegaly seems to exert a metabolically beneficial effect per se [19]. A recent metanalysis concerning the effect of PEG on glucose metabolism has shown a significant decrease in fasting glucose (FG), fasting insulin (FI), glycosylated haemoglobin (HbA1c), and the index of insulin resistance HOMA-IR during PEG monotherapy [20]. PEG as monotherapy has been proven to increase insulin sensitivity through the block of GH action at the receptor level and its consequent effect on insulin resistance and also to exert a positive impact on peripheral insulin sensitivity [19]. Probably, the removal of SRLs inhibitory effects on insulin secretion may have its role [19] since SRLs have been demonstrated to reduce insulin levels, to increase HbA1c and after-load glucose, with neutral effects on FG [19]. PEG addition to SRLs seems to mitigate SRLs effects on metabolism, thus producing an overall neutral metabolic effect [20]. Therefore, PEG as monotherapy or combined with SRLs seems to exert a direct positive impact on glucose metabolism, independent of disease control [20]. For this reason, according to the latest consensus on the management of acromegaly complications [22], PEG should be considered in patients with partial or complete resistance to first-line SRLs for whom glycemic control is challenging.

Differtenly, not univocal results were observed on lipid profile after PEG treatment, being reported both no change in lipid fractions and an increase in total (TC) and LDL cholesterol (LDL) with unchanged triglycerides (TG) and a significant decline in lipoprotein-A levels during PEG treatment [19]. Recently, a metanalysis about prospective studies reported an overall mild increase in TG and HDL after PEG treatment [20]. Nevertheless, the mechanism through which PEG can act on lipid metabolism is still unknown [19].

Furthermore, no significant changes in body mass index (BMI), systolic (SBP), and dyastolic (DBP) blood pressure have been found in acromegalic patients treated with PEG [8, 19, 20], albeit a significant decrease in metabolic syndrome (MetS) prevalence after a long-term therapy has been proven [13].

To date, scant data on efficacy, safety and metabolic impact of very prolonged PEG treatment are available. Therefore, the current retrospective study aimed at investigating the effects of 10-years PEG continuous treatment in patients with acromegaly, both in monotherapy or combined with SRLs, on disease control, maximal tumour diameter (MTD), gluco-insulinemic and lipid profile, and anthropometric parameters.

## Patients and methods

The current study consists in the retrospective analysis of data prospectively collected over the course of twenty years within the project 467-MET-9119-008, approved by local ethical committee in 2002.

### Inclusion and exclusion criteria

In this study were included adult patients with a diagnosis of acromegaly according to the following criteria: (1) GH > 2.5 µg/L as mean value of at least 3–6 samples drawn every 30 min over a period of 2 h; (2) GH nadir > 1.0 µg/L after a 75 g glucose load (in non-diabetic patients); (3) IGF-I above the normal range adjusted for gender and age [1, 23].

PEG therapy was started if the following characteristics were present: (1) resistance to long-term high-dose treatment with SRLs monotherapy, defined as IGF-I levels greater than 1.3 times the upper limit of normality (ULN) measured 28 days after the last SRLs injection after at least 6 months therapy at the highest available dose of SRLs; (2) patients on stable hormone replacement therapy for hypopituitarism for at least 6 months before study entry; (3) stable size of the pituitary tumour for at least 12 months before study entry, as documented by magnetic resonance imaging (MRI). Exclusion criteria were: (1) chronic hepatitis or liver failure; (2) suspicion of drug or alcohol abuse; (3) any other condition resulting in abnormal GH and IGF-I levels, such as severe hepatic or renal disease as well as malnutrition; (4) inability to self- administration the study drug; (5) women during fertile age with pregnancy desire; (6) treatment duration shorter than 5 years; (7) radiotherapy performed during treatment with PEG; (8) patients with incomplete data.

### Patients

**Fig. 1:**
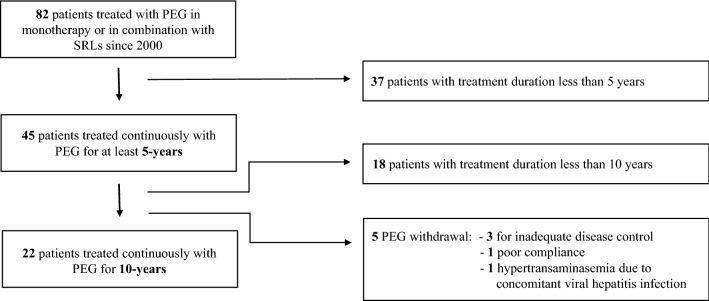
Selection of patients for the current study

Eighty-two consecutive patients underwent PEG therapy at the section of Endocrinology of University “Federico II” of Naples between January 2002 and Dicember 2021. Of these patients, 45 (19 men, 26 women, aged 46.8 ± 11 years) were treated with PEG at least for 5-years and were considered for this study (Fig. 1). Complete features at the study entry are shown in Table 1.

**Table 1:** Patient profile at study entry

Patients, *n*	45
Age, years	46.8 ± 11
Male/female, *n*	19/26
Microadenoma at diagnosis, *n* (%)	9 (20%)
Macroadenoma at diagnosis, *n* (%)	36 (80%)
Maximum tumour diameter, mm	17 ± 10.6
Empty sella, *n* (%)	8 (17.8%)
GH, ng/ml	19.9 ± 32.6
IGF-I, ng/ml	633.3 ± 285.5
IGF-I, x ULN	2.5 ± 1.2
Age at diagnosis, years	40.4 ± 11.4
Disease duration, years Previous therapy	6.4 ± 5.4
Neurosurgery, *n* (%)	31 (69%)
Second neurosurgery *n* (%)	4 (9%)
Radiotherapy, *n* (%)	1 (2.2%)
Octreotide, *n* (%)	19 (42.2%)
Lanreotide, *n* (%)	26 (57.8%)
SRLs + cabergoline, *n* (%)	14 (31.1%)

At diagnosis, nine patients (20%) harboured a GH-secreting pituitary microadenoma, and 36 (80%) a macroadenoma. Age at diagnosis was 40.4 ± 11.4 years, and disease duration before PEG treatment was 6.4 ± 5.4 years. Thirty-one patients (69%) underwent neurosurgery as first-line treatment, and four (9%) had undergone a second neurosurgery. Before starting PEG therapy, only one patient (2.2%) received pituitary radiation therapy. Previous medical therapy were lanreotide in 26 (LAN 57.8%) and octreotide LAR in 19 (LAR 42.2%); in 14 cases (31.1%) cabergoline (CAB) was add to SRLs to overcame the partial responsiveness to SRLs before the introduction of PEG.

### Study protocol

As for a retrospective study, we recovered the data of the patients from their charts. Our cohort of patients has been followed after the beginning of PEG therapy with a periodic control. As per internal protocol, at diagnosis and thereafter at 3–6 months intervals, all patients were admitted to the hospital for a complete physical, biochemical, and endocrine examination.

The present study considered three time-points: before starting PEG therapy, named baseline, after 5- and 10-years of PEG treatment.

Primary endpoints included: (1) proportion of patients with IGF-I normalization; (2) maximum tumour diameter (MTD) variation from baseline evaluated using MRI pituitary imaging; (3) proportion of patients with gluco-insulinemic and lipid metabolism abnormalities. Secondary endpoints included: (1) evaluation of IGF-I levels; (2) changes in FG, FI and derived index, and lipid fractions; (3) changes in anthropometric parameters.

At each time point, anthropometric parameters, including height, weight, BMI, SBP, DBP were recorded. Patients were classified as normal weight (BMI 18–24.9 kg/m^2^), overweight (BMI 25–29.9 kg/m^2^), and obese (BMI > 30 kg/m^2^) [24]. Systemic arterial hypertension was defined as SBP > 130 mmHg and DBP > 90 mmHg according to the guidelines of the American Society of Hypertension [25]. IGF-I levels were assessed in all patients at each visit, whereas GH levels were evaluated only at baseline due to the interference of PEG molecule in the assessment of this hormone.

Biochemical parameters, including fasting glucose (FG), fasting insulin (FI), and glycated haemoglobin (HbA1c), total cholesterol (TC), HDL, LDL, triglycerides (TG) were evaluated during this study. Based on glucose levels, the diagnosis of impaired glucose tolerance and diabetes mellitus was performed according to WHO guidelines [26]. Metabolic syndrome was diagnosed according to the National Cholesterol Education Program Expert Panel on Detection, Evaluation, and Treatment of High Blood Cholesterol in Adults (NCEP-ATP III) criteria [27]. Insulin resistance was assessed using HOMA-index in line with Matthews and coworkers [28], by calculating HOMA-IR = [FI (mU/l) × FG (mmol/l)]/22.5 as surrogate index of insulin resistance, and HOMA-β = [20 × FI (mU/l)/FG (mmol/l) –3.5] as surrogate index of insulin secretion [28], whereas ISI0 = 10,000/FI ($\mu$U/ml) × FG (mg/dl) was used to assess baseline insulin sensitivity [29].

Pituitary MRI with and without gadolinium was assessed before starting PEG and subsequently every year to evaluate pituitary adenoma dimensions and eventual change in size. For the current study, the MTD has been considered.

### Treatment protocol

According to the standard protocol of the centre [30, 31], SRLs were started at the initial dose of 20 mg/28 days for octreotide LAR (LAR) or 90 mg/28 days for lanreotide (LAN). Dose adjustment was carried out every 3–6 months based on serum GH and IGF-I levels. To normalize GH and IGF-I, SRLs dose was progressively increased. Thus, after long-term SRLs monotherapy (median 4 years, range 1–20 years), LAR dose ranged 30–40 mg/28 days (19 patients) and LAN dose ranged 120–240 mg/28 days (26 patients).

PEG was used as monotherapy in 6 patients (13.3%), and combined with SRLs in 39 (66.7%). PEG was administered via subcutaneous injections at a starting dose of 88.9 ± 77.1 mg/week (range 30–280 mg/week) in all the cohort, particularly 106.6 ± 42.8 mg/week in patients in monotherapy and 86.1 ± 81.1 mg/week in patients during combined therapy.

PEG starting dose was determined according to GH and IGF-I levels and patient characteristics, whereas SRLs dose was the same used before PEG addition in patients administered with combined therapy. Dose adjustment of PEG by ± 5 mg/day was carried out every 3 months for the first year, and every 3–6 months thereafter on the basis of IGF-I levels.

### Assays

Biochemical parameters were measured by standard methods. During the long study period, different IGF-I assays have been used; all IGF-I levels reported in the current study have been analysed according to the international standards. IGF-I is expressed in relation to the ULN to reduce the variability due to gender, age, and different assays.

### Statistical analysis

Data were analysed using SPSS Software for Windows, version 27 (SPSS, Inc., Cary, N.C., USA). Data are reported as mean ± SD, unless otherwise specified. The comparison between the numerical data before and after treatment was made by non-parametric Wilcoxon test for continuous variables. Non-parametric Mann–Whitney *U* test has been used for the comparison of numerical data between two different groups of patients as patients undergoing monotherapy vs combined therapy. The comparison between prevalence was performed by chi-squared test corrected by Fisher exact test when necessary. The correlation study was done by calculating Spearman’s correlation coefficients. Significance was set at 5%.

## Results

Clinical, metabolic, and hormonal parameters of the whole patient cohort at baseline and after 5- and 10-years of PEG therapy are shown in Table 2.

**Table 2:** Anthropometric, biochemical and hormonal parameters, PEG and SRLs dose before (baseline), after 5- and 10-years of PEG treatment

A–B *n* = 45 C *n* = 22	Baseline (A)	After 5-years PEG therapy (B)	After 10-years PEG therapy (C)	*p* (A vs B)	*p* (A vs C)	*p* (B vs C)
IGF-I, x ULN	2.5 ± 1.2	0.9 ± 0.37	0.99 ± 0.99	**< 0.0001**	**< 0.0001**	NS
Disease control (IGF-I < 1 x ULN), *n* (%)	0 (0%)	41 (91.1%)	20 (91%)	-	-	NS
Maximum tumour diameter, mm	17 ± 10.6	13.1 ± 9.1	14.4 ± 9.7	**0.01**	NS	NS
PEG, mg/week	88.9 ± 77.1	114.7 ± 67.7	131 ± 79	**0.001**	NS	NS
Monotherapy, A–B *n* = 6 C *n* = 4	106.6 ± 42.8	106.8 ± 90.4	88.7 ± 4.7	NS	NS	NS
Combined therapy, A–B *n* = 39 C *n* = 18	86.1 ± 81.1	119.3 ± 69.2	140.3 ± 83	**< 0.0001**	NS	NS
LAR, mg/28 days	30.0 ± 8.6	30.4 ± 6.4	32 ± 4.5	NS	NS	NS
LAN, mg/28 days	124.4 ± 51.8	129.7 ± 38.1	132.8 ± 48.1	NS	NS	NS
Fasting glucose, mg/dl	107.2 ± 18.8	115 ± 37.63	124.4 ± 45.6	NS	**0.03**	NS
HbA1c, %	6.1 ± 1.1	6.1 ± 1.2	6.1 ± 1.1	NS	NS	NS
Fasting insulin $\mu$U/l	15.5 ± 14.4	7.2 ± 5.6	9.7 ± 8.1	**< 0.0001**	NS	NS
HOMA-IR	4.4 ± 4.3	2.0 ± 1.5	3.3 ± 4	**< 0.0001**	NS	NS
HOMA-β	143.9 ± 124.7	77.1 ± 102.2	65.7 ± 33.3	**< 0.0001**	**0.01**	NS
ISI0	11.1 ± 8.1	23.9 ± 24.6	14.3 ± 6.7	**< 0.0001**	NS	**0.02**
Normal glucose tolerance, *n* (%)	16 (35.5%)	17 (37.8%)	6 (27.3%)	NS	NS	NS
Impaired Fasting Glucose, *n* (%)	16 (35.5%)	11 (24.4%)	5 (22.7%)	NS	NS	NS
Diabetes Mellitus, n (%)	13 (28.9%)	17 (37.8%)	11 (50%)	NS	**0.08**	NS
Total cholesterol, mg/dl	200.1 ± 32.1	191.6 ± 37.8	183.8 ± 34.5	NS	**0.03**	NS
LDL cholesterol, mg/dl	124.7 ± 31.2	116.5 ± 34.5	108.2 ± 34.5	NS	**0.05**	NS
HDL cholesterol, mg/dl	50.7 ± 14.3	49.7 ± 12.1	50.2 ± 14.9	NS	NS	NS
Triglycerides, mg/dl	123.2 ± 60.6	126.7 ± 73.8	141.7 ± 58.1	NS	NS	NS
Hypercholesterolemia, *n* (%)	24 (53.3%)	17 (37.8%)	10 (45.4%)	NS	NS	NS
Hypertriglyceridemia, *n* (%)	8 (17.8%)	11 (24.4%)	9 (41%)	NS	**0.04**	NS
Metabolic syndrome, *n* (%)	20 (44.4%)	17 (37.8%)	13 (59.1%)	NS	NS	NS
BMI, kg/m^2^	29.7 ± 4.8	29.4 ± 3.3	29.9 ± 4.5	NS	NS	NS
Normal weight, *n* (%)	3 (6.7%)	3 (6.7%)	2 (9%)	NS	NS	NS
Overweight, *n* (%)	24 (53.3%)	24 (53.3%)	11 (50%)	NS	NS	NS
Obese, *n* (%)	18 (40%)	18 (40%)	9 (41%)	NS	NS	NS
SBP, mmHg	129.1 ± 14	125.9 ± 13.2	136.9 ± 13.2	**0.02**	NS	**0.01**
DBP, mmHg	80.1 ± 8.3	84 ± 5.9	88.1 ± 9.9	**0.02**	**0.02**	**0.02**
Arterial hypertension, *n* (%)	15 (33.3%)	14 (31.1%)	12 (54.5%)	NS	NS	NS
AST, mg/dl	18.5 ± 7.6	22.3 ± 13.9	20.9 ± 11.8	**0.04**	NS	NS
ALT, mg/dl	21.1 ± 13.2	24.2 ± 12.6	20.3 ± 11.5	NS	NS	NS

The bold values mean statistically significant

NS not significant

### Baseline

At baseline, mean GH and IGF-I levels of all patients were 19.9 ± 32.6 ng/ml and 2.5 ± 1.2 × ULN, respectively. MTD was 17 ± 10.6 mm; secondary empty sella was present in 8 patients (17.8%).

Before starting PEG, abnormalities of glucose metabolism occurred in 29 patients (64.4%): impaired fasting glucose in 16 (IFG, 35.5%), and overt diabetes mellitus in 13 (DM, 28.9%). Patients with DM required medical treatment with metformin monotherapy in nine (69.2%), insulin in a basal-bolus schedule in one (7.7%), a combination of metformin and DPP-IV inhibitors in two (15.4%) and of metformin and insulin in one (7.7%). Particularly, FG was 107.2 ± 18.8 mg/dl, FI 15.5 ± 14.4 $\mu$U/ml, and HbA1c 6.1 ± 1.1%. Baseline GH significantly correlated directly with FI (*r* = 0.63, *p* = 0.002), HOMA-IR (*r* = 0.60, *p* = 0.004), HOMA-β (*r* = 0.54, *p* = 0.01), and indirectly to ISI_0_ (*r* = − 0.61, *p* = 0.004, Table 3). Similarly, baseline IGF-I significantly correlated directly with FI (*r* = 0.63, *p* = 0.002), HOMA-IR (*r* = 0.65, *p* = 0.001), HOMA-β (*r* = 0.43, *p* = 0.05), and indirectly to ISI_0_ (*r* = − 0.65, *p* = 0.001, Table 3).

**Table 3:** Correlation study between GH and IGF-I levels at baseline, disease duration, PEG dose and anthropometric and metabolic parameter

**Baseline**	BMI	SBP	ΔSBP	FG	ΔFG	FI	ΔFI	HOMA-IR	HOMA-β	ISI_0_	TC	TG	ΔTG	LDL	HDL	ΔHDL
Baseline GH	*r* = 0.07	***r* = **− **0.52**	***r* = 0.44**	*r* = 0.32	*r* = − 0.05	***r* = 0.63**	***r* = **− **0.52**	***r* = 0.60**	***r* = 0.54**	***r* = **− **0.61**	*r* = − 0.13	*r* = 0.04	*r* = − 0.13	*r* = − 0.02	*r* = − 0.23	***r* = 0.53**
	*p* = 0.77	***p* = 0.01**	***p* = 0.04**	*p* = 0.16	*p* = 0.81	***p* = 0.002**	***p* = 0.06**	***p* = 0.004**	***p* = 0.01**	***p* = 0.004**	*p* = 0.56	*p* = 0.87	*p* = 0.58	*p* = 0.92	*p* = 0.303	***p* = 0.01**
Baseline IGF-I	*r* = 0.17	***r* = **− **0.49**	***r* = 0.45**	*r* = 0.30	*r* = 0.07	***r* = 0.63**	***r* = **− **0.65**	***r* = 0.65**	***r* = 0.43**	***r* = **− **0.65**	*r* = − 0.17	*r* = 0.04	*r* = − 0.06	*r* = − 0.01	*r* = − 0.34	***r* = 0.43**
	*p* = 0.44	***p* = 0.02**	***p* = 0.03**	*p* = 0.17	*p* = 0.76	***p* = 0.002**	***p* = 0.01**	***p* = 0.001**	***p* = 0.05**	***p* = 0.001**	*p* = 0.45	*p* = 0.86	*p* = 0.78	*p* = 0.96	*p* = 0.12	***p* = 0.04**
Disease duration	***r* = 0.44**	*r* = 0.02	*r* = − 0.19	*r* = 0.30	***r* = **− **0.46**	*r* = 0.22	***r* = **− **0.54**	***r* = **− **0.56**	*r* = 0.13	*r* = − 0.20	*r* = − 0.20	*r* = 0.32	***r* = **− **0.45**	*r* = − 0.31	*r* = − 0.12	*r* = − 0.26
	***p* = 0.04**	*p* = 0.91	*p* = 0.38	*p* = 0.17	***p* = 0.03**	*p* = 0.34	***p* = 0.05**	***p* = 0.03**	*p* = 0.56	*p* = 0.38	*p* = 0.38	*p* = 0.14	***p* = 0.03**	*p* = 0.16	*p* = 0.60	*p* = 0.23

**10-years**	BMI	SBP	ΔSBP	FG	ΔFG	FI	ΔFI	HOMA-IR	HOMA-β	ISI_0_	TC	TG	ΔTG	LDL	HDL	ΔHDL
PEG dose	***r* = 0.51**	*r* = 0.07	*r* = 0.09	*r* = 0.10	*r* = 0.25	*r* = 0.06	***r* = **− **0.55**	*r* = 0.31	*r* = − 0.03	*r* = − 0.09	*r* = − 0.14	***r* = 0.55**	*r* = 0.31	*r* = − 0.07	*r* = − 0.28	*r* = − 0.17
	***p* = 0.01**	*p* = 0.74	*p* = 0.67	*p* = 0.64	*p* = 0.26	*p* = 0.83	***p* = 0.05**	*p* = 0.26	*p* = 0.92	*p* = 0.77	*p* = 0.55	***p* = 0.01**	*p* = 0.16	*p* = 0.75	*p* = 0.2	*p* = 0.45

Δ = percent change between baseline and 10-yaers evaluation. The bold values mean statistically significant

Dyslipidemia was found in 28 patients (62.2%): 20 (44.4%) with hypercholesterolemia, four (8.9%) with hypertriglyceridemia, and four (8.9%) with mixed dyslipidemia.

Normal weight, overweight, and obesity were found in three (6.7%), 24 (53.3%), and 18 patients (40%), respectively; BMI was directly related to disease duration before PEG therapy (*r* = 0.44, *p* = 0.04). Hypertension was found in 15 patients (33.3%), and 20 (44.4%) met the criteria for the diagnosis of MetS.

Concerning liver function, normal transaminases were registered in all patient before starting PEG treatment.

### Evaluation after 5-years of PEG treatment

**Fig. 2:**
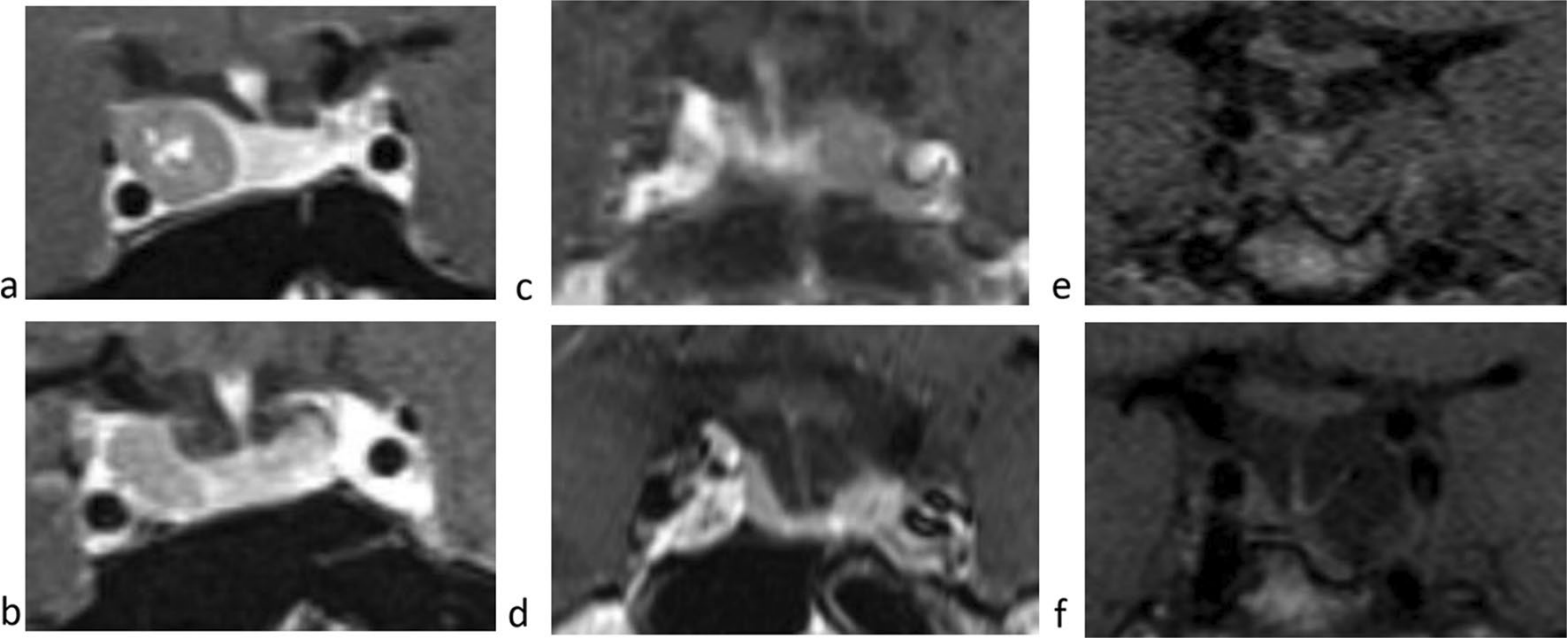
Pituitary MRI before (**a**), and after (**b**) 5-years therapy with PEG and SRLs combined therapy (patient not previously subjected to neurosurgery); before (**c**), and after (**d**) 10-years PEG monotherapy; before (**e**), and after (**f**) 10-years therapy with PEG and SRLs combined therapy

After 5-years of PEG, IGF-I levels were normal in 41 patients (91.1%), and a significant decrease in pituitary adenoma size compared to baseline (i.e. reduction of 25% in diameter) was found in 37% of patients. Except one case of monotherapy, all the cases of significant reduction in MTD were found in patients under combined therapy (Fig. 2a, b).

PEG dose was higher compared to the starting dose (*p* = 0.001), particulary in patients in combined therapy (*p* < 0.001), wheras no significant variation in SRLs dose was required.

Concerning glucose metabolism, although a slight increase in DM prevalence (37.8% vs 28.9%, *p = NS*) due to the increase in FG, HbA1c levels were stable compared to baseline. Noteworthy, all patients who developed DM were in combined therapy. On the other hand, a notable improvement in insulin profile was noted, since a significant decrease in FI, HOMA-IR, HOMA-β, and a significant increase in ISI_0_ were found (*p* < 0.001, Table 2). The surrogate index of insulin secretion HOMA-β was indirectly correlated to PEG dose (*r* = − 0.32, *p* = 0.04), whereas percent change (Δ) in FI was inversely correlated to IGF-I at baseline (*r* = − 0.59, *p* < 0.001). At 5-years follow-up, 13 patients (28.9%) still had DM and four (8.9%) were newly diagnosed with DM, requiring medical treatment with metformin monotherapy in 11 (64.7%), insulin in a basal-bolus schedule in two (11.8%), and a combination of metformin and GLP-1 agonists in one (5.9%), or metformin and DPP-IV inhibitors in one (5.9%) or triple therapy insulin, metformin and DPP-IV inhibitors in two (11.8%).

A slight decrease in TC, LDL and hypercholesterolemia prevalence was found. SBP was significantly reduced (*p* = 0.02) and DBP increased (*p* = 0.02), although no significant change in hypertension prevalence was registered.

After 5-years PEG therapy, ALT levels were stable compared to baseline, whereas a statistically but not clinically relevant increase in AST levels was found (*p* = 0.04). During the first 5 years of therapy, only two patients experienced a transient significant increase in transaminases (i.e. higher than 3 times the limit of normal). In these patients, PEG was maintained on therapy without dose escalation, performing at the same time a close monitoring until the transaminases values returned to normal limits. No patients experienced lipohypertrophy in the injection site.

Despite a comparable efficacy in term of disease control, when comparing mono- and combined therapy, a different metabolic impact was noted. Indeed, patients under monotherapy showed significantly lower FG (*p* = 0.01), FI (*p* = 0.008), HbA1c (*p* = 0.007), HOMA-IR (*p* = 0.001), and significantly higher ISI_0_ (*p* = 0.002) compared to patients in combined therapy. Conversely, patients under combined treatment showed significantly lower TC (*p* = 0.03), and LDL (*p* = 0.007) compared to patients in monotherapy.

### Evaluation after 10-years of PEG treatment

The evaluation after 10-years of PEG treatment was carried out in 22 of 45 patients (49%). Regarding the other 23 patients, PEG was withdrawn in five (11.1%), whereas 18 patients (40%) were in PEG therapy for less than 10-years at the time of the analysis (Fig. 1).

IGF-I levels persisted fully normalized in 91% of patients, whereas the remaining patients showed a partial disease control (i.e IGF-I <1.3 x ULN). MTD was stable compared to 5-years evaluation and no significant increase in MTD was registered. To note, all patients who showed a significant decrease in MTD during combined therapy were treated with LAN (Fig. 2e, f). MRI data were available only in three of the four patients receiving PEG monotherapy; a decrease in size was evident in two (Fig. 2c, d), whereas a stable dimension was assessed in the third.

PEG and SRLs doses were stable compared to 5-years evaluation. Higher PEG dose was required in young patients with elevated BMI as PEG dose was inversely correlated with age (*r* = − 0.45, *p* = 0.03), and directly to BMI (*r* = 0.51, *p* = 0.01, Table 3). Notable, patients requiring PEG dose above the median (120 mg/week) beared a significantly greater MTD compared to patients treated with PEG dose below (*p* = 0.04).

DM prevalence was slightly increased compared to baseline (*p* = 0.08), whereas glycemic control, expressed by HbA1c, was stable throughout the decade. At 10-years follow-up, 11 patients (50%) had DM, requiring medical treatment with metformin monotherapy in five (45.4%), insulin in a basal-bolus schedule in one (9.1%), and a combination of metformin and GLP-1 agonists in one (9.1%), or metformin and insulin in three (27.2%) or triple therapy insulin, metformin and DPP-IV inhibitors in one (9.1%).

Disease duration before PEG therapy was inversely related to ΔFG (*r* = − 0.46, *p* = 0.03). Compared to the beginning of therapy, HOMA-β, indicating insulin secretion, was significantly reduced (*p* = 0.01) compared to baseline, whereas the surrogate index of insulin sensitivity ISI_0_ persisted higher compared to baseline, although significantly reduced than 5-year assessment (*p* = 0.02). ΔFI was inversely related to baseline IGF-I (*r* = − 0.65, *p* = 0.01), PEG dose (*r* = − 0.55, *p* = 0.05), and disease duration before PEG (*r* = − 0.54, *p* = 0.05). Multiple regression analysis revealed that baseline IGF-I was the best predictive factor of ΔFI (*t* = − 3.61, *p* = 0.006).

Different impact on gluco-insulinemic metabolism of the two treatment regimens was reconfirmed even after 10-years, as a significantly lower FG (*p* = 0.03), and higher ISI_0_ (*p* = 0.04) were found in patients in mono- compared to the ones in combined therapy.

Concerning lipid profile, a significant decrease in TC (*p* = 0.03) and LDL (*p* = 0.05) compared to baseline was found, being PEG dose correlated to TG (*r* = 0.55, p = 0.01).

Compared to baseline, DBP (*p* = 0.02) was significantly increased, with a concomitant increase in hypertension prevalence.

Transaminases levels were stable compared to both baseline and 5-year assessment (Table 2), and no case of lipohypertrophy in the injection site was registered during a decade of PEG therapy.

## Discussion

The results of the current study demonstrated that 10-years continuous PEG treatment results in disease control in almost all acromegalic patients resistant to SRLs, with a prolonged beneficial impact on insulin, and lipid metabolism, without tachyphylaxis and negative effects on tumour growth.

Previous studies have shown that long-term PEG therapy improved disease control over time, both in monotherapy and combined with SRLs [10–17]. Concordant results on long-term efficacy were found analysing data from registered study ACROSTUDY [10, 16], and spontaneous studies on smaller cohort in single centre experiences [10, 12–16]. Noteworthy, most studies considered pooled data, thus the extrapolation of data on continuous therapy for 10-years is not possible. In this context, the value of the current study is the high number of patients treated continuously with PEG for 10-years in a single referral centre. Previous studies reported normal IGF-I in about 70–90% of patients treated with PEG for at least 5-years [10–17]. Although considering PEG long-term monotherapy, a slightly lower prevalence of disease control was found (67.5%) [11]. In the current series, IGF-I was fully normalized in 91% of patients after 5- and 10-years of PEG, either in mono- or combined therapy; a prevalence fairly higher than previous results. Probably, the strict monitoring together with the use of high PEG doses (median weekly dose was 105 mg at 5-years and 120 mg at 10-years evaluations) allowed to achieve such an encouraging result.

Previous evidences have demonstrated that tumour growth during PEG therapy could be a major concern. Despite in most cases the dimension of the tumour remains unchanged or even it is possible to observe a decrease in size, pituitary tumour volume increase has been shown to occur in approximately 7% of patients during PEG therapy [6]; even rarely many years after the beginning of therapy [11]. This increase in size seems to be ascribable to SRLs withdrawal or to the natural history of an aggressive tumour, rather than a direct effect of PEG therapy [17]. In our cohort, MTD was significantly reduced after 5-years, remaining stable at the subsequent 10-years evaluation, without any case of significant increase in MTD registered in our series. Of note, a decrease in size was found after 10-years monotherapy in one patient (Fig. 2b), suggesting that natural history of the tumour rather than PEG itself mostly influences change in tumour size.

Over the last three decades, discordant results have been obtained studying the impact of SRLs on glucose homeostasis. Increasing evidence has demonstrated an overall modest and transient negative impact [30, 32–41], confirmed by a recent meta-analysis [21], in which SRLs have been reported to reduce insulin levels and increase HbA1c and after-load glucose, with neutral effects on FG. Conversely, another study [38], investigating the metabolic effects of first-line SRLs or surgery in a large series of acromegalic patients, has demonstrated SRLs to reduce FG, HbA1c, and DM prevalence with disease control, suggesting that improvement in glucose and insulin profile is mainly ascribable to obtaining disease control [38]. Concerning the effect of PEG monotherapy on glucose metabolism, a recent metanalysis [20] has shown a significant decrease in FG, FI, HbA1c, and the index of insulin resistance HOMA-IR; appearing this beneficial role to be independent of disease control. On the other hand, the addition of PEG to SRLs seems to mitigate SRLs actions on metabolism, producing an overall neutral metabolic effect [20]. Results of the current study are consistent to previous since a different metabolic impact has been noticed when comparing mono- and combined therapy. After 5-years, patients on monotherapy experienced a significant greater decrease in FG, FI, HbA1c, and HOMA-IR compared to those on combined therapy. Monotherapy beneficial impact on gluco-insulinemic metabolism was maintained even after 10 years as significantly lower FG, and higher insulin sensitivity index ISI_0_ where found when compared to combined therapy. Furthermore, substantial neutral effect on glycemic status of combined therapy was confirmed in this study. Although there was an increase in FG, and consequently in DM prevalence, HbA1c remained stable during a decade. HbA1c stability for a decade represents an important outcome, considering that acromegalic patients are at-risk population for DM and 64.4% of patients had already an impairment in glucose profile before starting PEG, particularly IFG in 35.5% and DM in 28.9% of patients. Furthermore,  mean age at the last evaluation was 55.5 years, being reported an increased risk of DM at this age also in the general population. This hypothesis seems to be supported by concomitant decrease in HOMA-β, a derived index of insulin secretion, probably due to the physiological reduction of insulin secretion consequent to the aging process. Noteworthy, patients who developed DM had IFG prior to PEG beginning and were all on combined therapy. Furthermore, to the knowledge of the authors, the current study firstly demonstrated that ΔFI was inversely related to baseline IGF-I, and that this IGF-I value was the best predictive factor of ΔFI. Therefore, baseline IGF-I seems to be able to predict the degree of insulin reduction, and consequently the improvement of the related indices. Notably, this is the first study demonstrating that disease duration before PEG starting inversely correlated with the improvement in gluco-insulinemic profile and the insulin resistance index HOMA-IR while on PEG therapy, supporting that an early switch to PEG in patients with proven resistance to SRLs therapy allows to achieve a greater metabolic benefit, being crucial in a patient already showing a pre-existing impairment in glucose metabolism.

Concerning lipid profile, the mechanism through which PEG can act on lipid metabolism is still unknown [20]. Previous studies reported non-univocal results about the impact of PEG on lipids since both no change in lipid fractions, and increase in TC, LDL, with unchanged TG have been found [19]. Recently, a metanalysis about prospective studies reported an overall mild increase in TG and HDL after PEG treatment [20]. To the authors knowledge, this is the first study investigating the effects of 10 years of continuous PEG therapy on lipid metabolism. An overall improvement in total cholesterol and particularly a significant decrease in LDL cholesterol (i.e., atherogenic) was seen after 10 years, with a greater beneficial effect of combined therapy on lipid metabolism as compared to monotherapy, thus suggesting that patients might benefit from additive effects of SRLs [42] to those of PEG on lipid fractions. Albeit not significantly, long-term PEG resulted in an increase of circulating triglycerides, as previously reported [20, 43]. Interestingly, PEG dose was directly correlated with triglyceride levels. This innovative finding might be ascribed to several factors. First, the role of dietary habits cannot be excluded, although no significant change in body weight and BMI was seen throughout the study, consistent with previous reports [13, 44]. At total body magnetic resonance imaging, a significant increase in visceral adipose tissue of 187% and of intrahepatic lipid (from 1.75 to 3.04%) has been documented following long-term PEG treatment [44], suggesting a direct effect of this compound not necessarily limited to the inhibition of IGF-I secretion. By mimicking the effects of endogenous GH excess, PEG might further increase GH-dependent lipolysis, thus leading to an increase in TG levels. In this light, the higher the PEG dose used to control IGF-I excess, the higher the circulating TG levels as seen in the current study. Furthermore, data of our study were in line with previous findings, since no changes in BMI during PEG therapy were found [8, 19, 20]. Differently to previous evidences, SBP, DBP, and hypertension prevalence has been found to be increased after 10-years. Patients’ follow-up was shorter in previous studies, suggesting that the increase in hypertension prevalence could be prior ascribed to the aging process in an at-risk population rather than a direct therapy effect.

Altogether, the results of the current study demonstrate that the use of PEG produces a beneficial impact on metabolic profile, most likely mediated by the achievement of biochemical control.

## Conclusion

After 10-years continuous therapy, PEG is effective in disease control and safe, as both mono- and combined therapy. No significant increase in pituitary tumour dimension has been registered in this study, whereas a great number of patients achieved a significant reduction in tumour size within the first five years of treatment, which was maintained during the subsequent years. Although mono- and combined therapy showed a different impact on glucose and lipid metabolism, an overall beneficial impact on both persists after prolonged therapy. As the extent of the metabolic improvement has been demonstrated to be inversely related to disease duration before PEG, this treatment should be promptly started in patients resistant to SRLs. Thus, in our opinion, the present study provides new insights into current knowledge about the long-term efficacy and safety of PEG and may support the clinical management of acromegalic patients during PEG therapy, to offer a tailored therapy based not only on long-term biochemical and tumoral efficacy but also on the metabolic impact of this treatment.

## Data Availability

The datasets generated during and/or analysed during the current study are available from the corresponding author on reasonable request.
